# Pitfalls in diagnosing a small cystic insulinoma: a case report

**DOI:** 10.1186/1752-1947-1-181

**Published:** 2007-12-17

**Authors:** Mirjana Sumarac-Dumanovic, Dragan Micic, Miodrag Krstic, Maja Georgiev, Aleksandar Diklic, Svetislav Tatic, Danica Stamenkovic-Pejkovic, Aleksandra Kendereski, Goran Cvijovic, Aleksandra Pavlovic

**Affiliations:** 1 Institute of Endocrinology, Diabetes and Diseases of Metabolism, University Clinical Centre, Belgrade, Serbia; 2 Institute of Digestive Diseases, University Clinical Centre, Belgrade, Serbia; 3 Institute of Pathology, Belgrade Medical School, Belgrade, Serbia

## Abstract

Insulinoma is a rare pancreatic endocrine tumour and is typically sporadic and solitary. Over 90% of all insulinomas are benign. Cystic insulinomas are also rare. It is not difficult to determine the site of such neoplasm, as cystic insulinomas are usually 4–10 cm in diameter. We present the case of a patient with a histologically confirmed cystic insulinoma diagnosed after approximately 10 years of hypoglycaemia symptoms. This case is unique because of the small size (2.2 cm) of the tumour. Endoscopic ultrasound (EUS) was useful for localizing this tumour.

## Introduction

Pancreatic endocrine tumors are rare lesions, with a reported incidence of four cases per 1 million patients a year [1]. Of these lesions, insulinomas are the most common. The majority of patients diagnosed with an insulinoma are between 30 and 60 years of age, with women accounting for 59 % of cases [2,3]. Most insulinomas are sporadic in their origin. They are more likely to be multiple in patients with multiple endocrine neoplasia type I [1,4]. Pancreatic neuroendocrine tumors rarely manifest cystic changes [5]. Cystic neuroendocrine tumors are difficult to diagnose preoperatively because the majority of these tumors are non-functional, and computerized topography (CT) does not differentiate these tumors from other cystic neoplasms. Cystic neuroendocrine tumors represent a subgroup of pancreatic cystic and neuroendocrine tumors with malignant potential. Their high resectability rate further supports the role of surgical exploration and resection in the treatment of a pancreatic cystic neoplasm [6]. Insulinoma tumors are often difficult to detect as the symptoms largely precede occurrence of a visualized tumor [3]. Cystic insulinomas are rare, with only a few cases having been reported in the literature [6].

In our case report we point out the difficulties in diagnosing a small cystic insulinoma. Diagnosis of insulinoma could be difficult if the functional activity is incomplete, possibly leading to blunted symptoms of hypoglycemia. Our case shows the usefulness of endoscopic ultrasound for localizing a small cystic tumor from other pancreatic lesions.

## Case presentation

A 51-year-old male (BMI 27.5 kg/m^2^) was admitted to hospital due to recurrent episodes of confusion, light-headedness, chills, palpitations and shakiness for more than eight years. He typically experienced these symptoms after extensive physical activities.

The patient's past medical history indicated hypertension and he was taking an ACE inhibitors. There was about a history of alcohol abuse in the past. There was no family history of hyperparathyroidism, ulcer disease or hypoglycaemia, but his father had had hypertension.

He underwent a 72 hour-fast test, interrupted after 36 hours due to neuroglycopenic symptoms (plasma glucose 2.4 mmol/l, insulin 21.1 mU/l (n.r. 1–20 mU/l), C-peptide 1.5 nmol/l (n.r. 0.3–0.7 nmol/l)). During the course of the fasting his blood was checked with GC-MS (Gas/Mass Chromatography) for oral hypoglycaemic drugs and was positive on tolbutamide. Both he and his family denied any intake of oral antidiabetic preparations, but there were no further hypoglycaemia attacks in subsequent days in the hospital.

One year later, during a second episode of hospitalization, the test on tolbutamide during the *72 hour-fast *was repeated and the result was negative. This time the fasting was interrupted after 8 hours (plasma glucose 1.8 mmol/l, insulin 16.3 mU/l). C-peptide suppression test [7] showed good suppression of C-peptide (46%). Abdominal ultrasound, magnetic resonance imaging (MRI) and EUS were negative. He refused surgical exploration of the pancreas and on that occasion he was prescribed diazoxide. He started the treatment but terminated it after a while on his own accord. In the meantime between the second and the third episodes of hospitalization three years later, he experienced hypoglycaemia symptoms with similar frequency and his health insurance sent him back to hospital for further assessment after a car incident.

**Figure 1:**
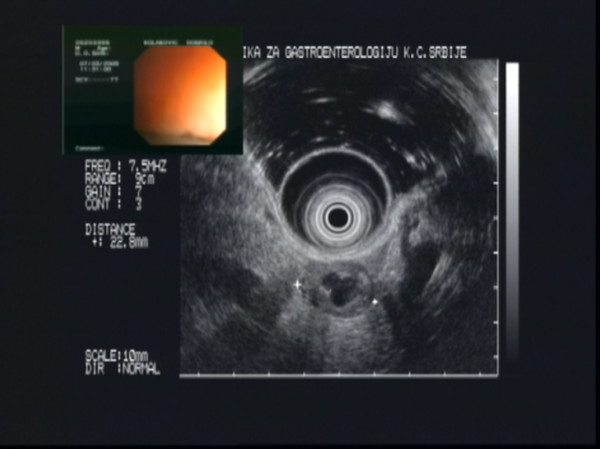
Endoscopic ultrasonography showing a cystic tumour in the pancreatic body (Olympus GIF-130 video echo-endoscope with 7.5/12 MHz switchable radial probe).

During his latest hospital visit, a 72 hour-fast test was interrupted on the first day after 5 hours (glucose 1.5 mmol/l, insulin 31.4 mU/l, C-peptide 2.1 nmol/l). This time C-peptide suppression test was in favour of autonomous insulin secretion: 30 minutes 3.53% suppression, 60 minutes 3.83% suppression, 90 minutes 20% suppression. MRI was again negative. A selective pancreatic arteriography showed a focal avascular lesion in the body of the pancreas near the tail. EUS confirmed a lesion in the pancreatic body near the tail and no other lesions. It was a cystic lesion measuring 2.28 cm in diameter with a very thick wall measuring 3–4 mm (Figure 1).

**Figure 2:**
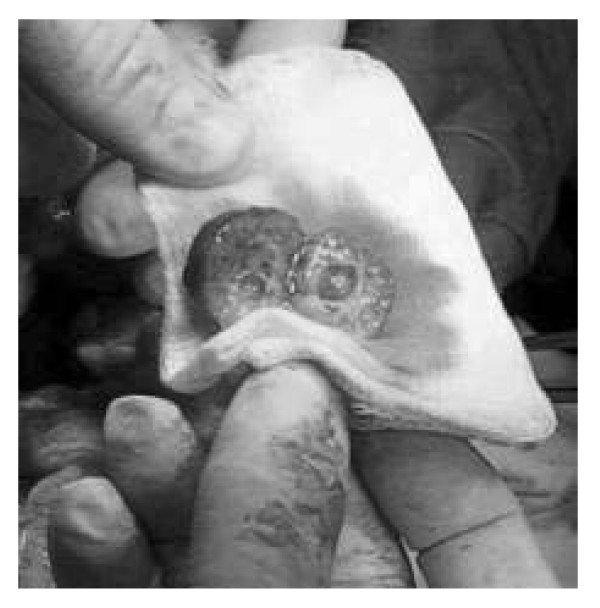
Postoperative finding: 2.5 × 2 × 2 cm well bounded tumour, weighing 4 grams, cystic formation of 8 mm in diameter in the middle of the tumour.

**Figure 3:**
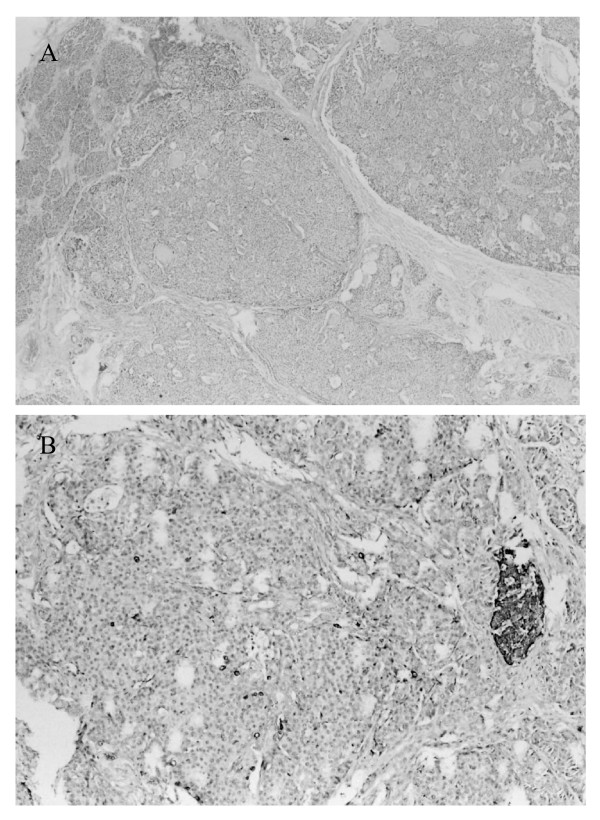
Insulinoma. Acini of exocrine pancreas (in the upper left corner), Haematoxylin-eosin, 60× (A). Insulinoma. Chromogranin A, 200× (Mild to moderate immunopositivity generally and scattered cells with intense immunopositivity. Strongly immunopositive cells of islet of Langerhans in the surrounding tissues) (B).

He was operated on and a 2.5 × 2 × 2 cm well bounded tumour, weighing 4 grams, was removed from the pancreas body (Figure 2). There was no evidence of gross invasion, abnormal lymph nodes or liver metastases. Pathological evaluation revealed a well differentiated insulinoma with fibrous connective tissue and cystic formation of 8 mm in diameter in the middle of the tumour. The tumour consisted of small nests of homogeneous, cylindrical tumour cells without any cytological atypia, mitotic activity or necrosis. Immunohistochemical staining confirmed the diagnosis of insulinoma (Fig 3). Seven days after the operation, the patient was discharged with normal glucose profiles. Ten months after the operation the patient is still free of the previous symptoms.

## Discussion

This is a report of a patient with an unusual course of disease which was contributed to by a falsely positive test for tolbutamide probably due to an insufficiently precise method used to determine the presence of sulphonylurea medications. It is also likely that the chronic course of this disease is the consequence of the small size of the tumour. Insulinoma tumours are often difficult to detect as the symptoms largely precede occurrence of a visualizable tumour [8]. In the case of this patient, all three fasting tests were positive although the time of the interruption was shortened over time. C-peptide suppression test could have been helpful but in our case on two occasions we obtained two different results [7]. Cystic endocrine tumours of the pancreas rarely occur, and only a few cases of cystic insulinomas have been reported to date [9]. Diagnosis of insulinoma could be difficult if the functional activity is incomplete, possibly leading to blunted symptoms of hypoglycaemia and failure of laboratory investigations to provide evidence of hyperinsulinemia [8]. A clinical case of cystic insulinoma was recently reported by histological examination after surgery, characterized by a high intracystic insulin concentration despite normal blood basal levels of the hormone [10]. In that case it was suggested that cystic formation within a solid endocrine neoplasm may be due to haemorrhage and necrosis of tumour cells with disruption of tissue planes, leading to cyst development [11] or that these slow-growing tumours develop a fibrous capsule, which eventually decreases the blood supply to the tumour leading to infarction and liquefaction necrosis [11]. The evolution of cysts can occur in small tumours and suggests that haemorrhage may be the inciting event.

Generally ultrasound (US), CT, angiography and transhepatic portal venous sampling (THPVS) have been widely used in the preoperative localisation of such tumours with various rates of accuracy of localisation reported by investigators [12]. The results of non-invasive-imaging techniques, in general, have been discouraging. Sensitivities ranging from 9 to 63% and from 16 to 72% have been reported for US and CT scanning, respectively [12]. Higher sensitivity (ranging from 36 to 91%) has been reported for angiography [12]. The best results have been obtained by THPVS along the pancreatic vein: a sensitivity of 82% and a specificity of 91% were reported by Vinik [13]. Some centres use preoperative endoscopic ultrasound which has reported accuracy rates of 60–90% [14]. Lesions in the tail of the pancreas may be missed using endoscopic ultrasound; however, these lesions are usually easily identified intraoperatively [14]. Approximately, 40% of all insulinomas are not localised preoperatively, and between 3 and 10% remain occult even after intraoperative palpation and the use of intraoperative ultrasound (3,4). Portal venous sampling was not necessary preoperatively, even in the case of occult insulinoma. This invasive technique, although helpful, cannot give precise anatomical localisation and indicates only the region of the pancreas from which the excess insulin secretion emanates [13]. Localisation of an insulinoma with laparoscopic ultrasonography has also been reported [15].

Some authors consider endoscopic ultrasonography (EUS) to be the single most important preoperative localisation study needed [15]. EUS allows high resolution imaging of the pancreas [15]. It is accurate for pre-operative localization of pancreatic neuroendocrine tumours, mainly insulinomas, and it is a good alternative to other more invasive methods. The images of the inner structure of cystic lesions that this modality provides are not only more accurate, but also displayed in fine detail [14].

However, the differential diagnosis of cystic pancreatic lesions by EUS is still very difficult. Although fasting tests confirmed autonomous insulin secretion in our patient, angiography finding of a vascular area in pancreas did not indicate that the EUS visualized cystic tumour in the pancreas was an insulinoma.

## Conclusion

The differential diagnosis of cystic pancreatic lesions by EUS is still very difficult. Although fasting tests confirmed autonomous insulin secretion in our patient, the angiography finding of a vascular area in the pancreas did not indicate that te EUS visualized cystic tumour in the pancreas was an insulinoma. In our case report we point out the difficulties in diagnosing a small cystic insulinoma if the functional activity is incomplete, possibly leading to blunted symptoms of hypoglycaemia. As far as we know this is one of the few reported cases of a small cystic insulinoma. Our case shows the usefulness of endoscopic ultrasound for localizing small cystic pancreatic tumors.

## Abbreviations

EUS: Endoscopic ultrasound; 

MRI: Magnetic resonance imaging.

## Competing interests

The author(s) declare that they have no competing interests.

## Authors' contributions

MSD made substantial contributions to conception and design, or acquisition of data, or analysis and interpretation of data. DM gave final approval of the version to be published. MK performed EUS and had been involved in drafting the manuscript. MG conceived the study, and participated in its design and coordination and helped to draft the manuscript. AD performed pancreatic operation. ST performed histological finding. DSP conceived of the study, and participated in its design and coordination and helped to draft the manuscript. AK conceived of the study, and participated in its design and coordination and helped to draft the manuscript. GC conceived of the study, and participated in its design and coordination and helped to draft the manuscript. AP performed EUS and was involved in drafting the manuscript.

## Consent

The patient gave written informed consent for publishing his data as case report.
